# Employing the equity lens to understand multisectoral partnerships: lessons learned from a mixed-method study in Canada

**DOI:** 10.1186/s12939-022-01746-w

**Published:** 2022-09-26

**Authors:** Suvadra Datta Gupta, Vaidehi Pisolkar, Jacob Albin Korem Alhassan, Allap Judge, Rachel Engler-Stringer, Lise Gauvin, Nazeem Muhajarine

**Affiliations:** 1 Department of Community Health and Epidemiology, University of Saskatchewan, Saskatoon, Canada; 2 Saskatchewan Population Health Evaluation and Research Unit (SPHERU), University of Saskatchewan, Saskatoon, Canada; 3 Department of Social and Preventive Medicine, Université de Montréal, & Centre de Recherche du Centre Hospitalier de L’Université de Montréal, Montréal, Québec Canada

**Keywords:** Equity, Health equity, Multisectoral partnerships

## Abstract

**Background:**

Multisectoral approaches to health are collaborations between stakeholders across multiple sectors, usually formed to address issues that affect health but go beyond the purview of one particular sector. The significance of multisectoral partnerships to attain health equity has been widely acknowledged. However, the extent which equity can be attained depends upon the perceptions of various stakeholders. We examine how multisectoral partnerships promoting healthy eating and active living conceptualized and employed an equity lens in their work.

**Method:**

This study is part of a larger pan-Canadian mixed-method research and knowledge sharing program entitled MUSE (Multisectoral Urban Systems for health and Equity in Canadian cities). Data collected from both quantitative and qualitative sources for two sites of the MUSE project-Saskatoon and Toronto were analyzed. In the qualitative part, 30 semi-structured key informant interviews were conducted with key stakeholders from six different multisectoral partnerships based in Saskatoon and Toronto. Data were analyzed in an inductive way. In the quantitative part, a survey with 37 representatives of stakeholder organizations was carried out. Simple descriptive statistics (means and percentages) were used to observe the distribution of data and to complement the qualitative analysis.

**Results:**

Equity was not a central component in program design although participants addressing equity, did so by discussing accessibility. How much consideration was given to equity varied as a function of the type of partnership. Most participants emphasized geographical accessibility but a few mentioned financial accessibility. Collaborative leadership style facilitated a participatory decision-making process, and thereby upholding equity in the partnership decision-making process. Communication, networking, and negotiation skills were found to be core competencies of a leader that contributed in upholding equity in partnership dynamics. The study also showed some challenges to embed equity in partnership works, such as the lack of comprehensive understanding of population health and its equity tenet.

**Conclusions:**

Findings indicate that multisectoral partnerships aimed at promoting healthy eating and physical activity experience several challenges to attain equity within the partnership as well as in the partnership-based works aimed at reducing health equity in populations. Factors identified can support decision makers commit to and work to attaining equity within their partnerships as well as in the partnership-based work in the community and beyond.

## Background

In an influential article on equity and health, Margaret Whitehead conceptualized health inequities as disparities in health status that are “not only unnecessary and avoidable but, in addition, are considered unfair and unjust” [1]. Since then, the concept of equity has greatly evolved even though adherence to human rights and social justice principles have remained a central tenet [2, 3]. Health inequity means that there are systematic differences in accessing opportunities to attain maximum health potential which can be avoided [3], and, therefore, it is closely linked with the social determinants of health and the systems that influence their unequal distribution [4]. The complex and dynamic nature of these factors requires multisectoral collaboration between public health and other sectors to shape policy; a key strategy iterated in the Rio declaration on Environment and Development as well as in the Health in All Policies (HiAP) movement [5, 6].

Multisectoral approaches to health are coordinated actions from stakeholders across sectors, constituencies, and communities at large usually formed to address complex health issues that go beyond the capacity or responsibility of any single sector [7]. For example, according to recent research findings, improvements in several health indicators such as reduction in maternal and child mortality were due to improvements in key areas outside of the health sector such as income generation and improved literacy [8]. Recognition of the significance of multisectoral actions to improve health and well-being in population dates back to the historical Alma-Ata declaration and subsequent health promotion movement of the 80 s [9]. Although the terms ‘multi’ or ‘intersectoral actions’ were not explicitly mentioned, the Alma-Ata declaration emphasized collaborative actions between multiple sectors to be key in attaining health equity [10]. This, however, met with some critical challenges, e.g., difficulty to reach a shared vision of health equity, and resistance from non-health sectors to embed health equity in non-health policies [10]. Nonetheless, multisectoral partnerships continued to be an essential component in numerous international conferences and policy consultations on health promotion such as the Ottawa Charter for Health Promotion as well as the Health in All Policies (HiAP) [10, 11]. The 2017 World Health Organization (WHO) global action plan to promote physical activity endorsed 20 policy actions, which included strengthening multisectoral partnerships as a way of crafting and strengthening active systems in society, making equity a cornerstone in the policies [12]. In 2018, the Astana declaration reaffirmed the commitments made in the Alma-Ata declaration, reiterated the significance of health equity, and presented multisectoral partnerships as a key component to attain well-being for all [13, 14].

However, despite efforts to re-orient the health system towards an equitable one, a significant health equity gap persists within and between countries. In the United States, only 10% of the public health targets of Healthy People 2010 were achieved, and the poor performance was attributed to a lack of collaboration, shared responsibilities, and understanding of multisectoral partnerships [15]. In Ontario, Canada, although provincial health standards required public health units to address the existing health inequalities through actions taken on social determinants of health, public health units varied in their understanding of and capacity to apply this concept in practice [16]. In order to shed further light on the process of addressing healthy equity in the context of multisectoral partnerships, we examine how stakeholders from six multisectoral partnerships located in Toronto and Saskatoon conceptualized and applied the concept of equity in their work and functioning. Given the significance of multisectoral partnerships to advancing health equity, our study advances knowledge about how to apply the health equity lens necessary for achieving better collaboration within multisectoral partnerships.

## Materials and methods

This study is part of a larger pan-Canadian research and knowledge sharing program entitled Multisectoral Urban Systems for health and Equity in Canadian cities (MUSE). MUSE is a mixed-method program of research involving surveys, key informant interviews, focus group discussions, and document reviews with the purpose of gathering evidence for informed practice of multisectoral partnerships to achieve health outcomes by promoting active living and healthy eating. The present study analyzed data collected from both quantitative and qualitative sources for two sites of the MUSE project—Saskatoon and Toronto. The project was formed to promote health and equity across Canadian cities.

### Data collection

In the qualitative part of this study, 30 semi-structured key informant interviews were conducted with key stakeholders representing six different multisectoral partnerships^1^ located in Saskatoon (*n* = 16) and Toronto (*n* = 14). Participants in these interviews were representative of the composition of the partnerships and had worked for different organisations such as municipal government, public health organizations, academia as well as community-based organizations. Interviews were conducted by two Research Assistants (one male and one female) and one Research Officer (female) of the MUSE research team. Prior to the interview, the researchers contacted participants through email and introduced themselves, informed their research interests, the study objectives, and requested a suitable time and place to hold the interview. Most interviews took place at participants’ workplaces. The study followed a purposive sampling strategy. Researchers reached out to the people who worked with the multisectoral partnerships since initiation, were working with them at the time of the study, and in some cases also reached out to people who were suggested by other participants. All the interviews were done in person. Most interviews took approximately 1 h. The researchers had an interview checklist and necessary prompts were made throughout the interview to gain more insight into participants’ responses. Topics discussed during the interview included: participants role during partnership initiation, their current role at the MP, monitoring, and evaluation of MPs, how members were brought into partnerships, discussions on ‘equity’ among partners, and barriers and facilitators to success.

A survey with representatives of organizations was carried out online from February 02 to May 13, 2020 and the total number of participants was 37. The purpose of the quantitative survey was to collect complementary information about the multisectoral partnerships from the perspective of stakeholders. The anonymous questionnaire consisted of three survey instruments namely, the Wilder Collaboration Factors Inventory [17], the Self-evaluation Tool for Action in Partnership (SETAP) [18], and an instrument about whether or not the recommended evidence-based policy targets for physical activity and healthy eating [12] were pursued in the work of the partnership (these latter data are not reported in this paper). The Wilder tool assesses factors influencing the success of partnerships such as the characteristics, process, and resources of the partnerships. SETAP measures equity in partnership dynamics by examining whether or not equal importance and acknowledgment are given to all points of view, benefits are distributed equally, and if partners have accountability.

### Data analysis

All key informant interviews were audio-recorded and transcribed verbatim. Four research assistants coded the data. Data were entered in NVivo and analyzed in an inductive way. A coding framework was developed based on multiple reviews of the dataset that helped with organizing the data in a systematic way into meaningful categories. Codes and categories were reviewed multiple times to identify recurring themes. Data were scrutinized to identify participants’ perspectives on employing a health equity lens in the work of the multisectoral partnerships. Data across the two cities were pooled together.

### Theoretical framework

Literature on multisectoral partnerships has adopted a range of frameworks that differ based on their purpose, components, and the level of the ecosystem [7]. Willis et al. show that although some frameworks focus on the context, formation, and challenges of partnerships, others focus on inter-organizational processes such as planning, collaboration as well as strategies to map the effectiveness of partnerships [7]. Given our broad interest in health equity and the need to adopt a broad perspective, we adopted the conceptual framework for assessing health equity action in public health developed by Lambton Public Health [19]. This framework synthesizes multiple dimensions of public health actions into two broad components that drive organizational capacity to attain health equity [19]. Internal drivers of the framework denote elements that are internal to the organizations such as leadership, informal and formal systems, and external drivers are those that are external to the organizations such as political will, public support, etc. [19]. The framework is useful to recognize how public health leaders understand and embed equity in the work of multisectoral partnerships. We thus examined how stakeholders in multisectoral partnerships perceived equity, and how equity had been upheld in the processes and functioning of the multisectoral partnership itself. Furthermore, we explored factors such as leadership styles, construction, and prioritization of equity in partnerships as well as challenges to achieve equity which are elements within the internal driver component of the Lambton framework.

## Results

### Qualitative study findings

The Qualitative part of the study details how equity was understood and applied in work based on partnerships. While reflecting on their understanding and experiences, most participants indicated that discussion on equity was important. However, most of them framed equity strictly around accessibility. The framing of equity influenced how much priority equity was given in partnership-based work. Style of leadership is a key factor in attaining equity in partnerships, and certain leadership qualities (discussed below) were found to be conducive to the success of multisectoral partnerships. Apart from that, the gap between organizational goal and the goal of multisectoral partnership set challenges to embed equity in the partnership-based work.

#### Framing of equity

Participants framed equity around accessibility. Most participants emphasized geographical accessibility, i.e., reaching people and neighbourhoods through transit services or designing bike lanes. In participants’ view, transit revealed the essence of equity by making roads accessible to everyone. Participants from a transit focused partnership mentioned weighing inequity in decision making by considering ‘accessibility of transit stops’ and ‘people with disabilities’ while re-evaluating the transit route. As one of the participants noted:

*“We just tried to get a very wide cross-section, recognizing that there are a lot of users of this street and some of them are driving, but a lot of them are taking [public transit], a lot of them are walking. Some of them have disabilities, so we have to take into account accessibility, too, and are we providing a good environment for people to get around whether they’re walking or in a wheelchair or whatever. That was a key consideration”*

Another participant noted:

*“There was definitely consideration of equity…Because it was so transit focused, beyond that scope, it wasn’t included”*

However, some participants mentioned the financial aspect of equity as well. As the participant noted:

*“But what it did is it [the program] often just gave the recipient the confidence to go and seek employment and in terms of the relatively low cost of the intervention had some fabulous results in terms of employment outcomes. So that we thought was really core public health”*

#### Prioritization of equity

Whether equity was a priority in program design and implementation, depended upon the underlying issue the MP was trying to address. In most cases, equity was not an essential or explicit component in priority setting at the time of initiation of MP. This is particularly true for MPs that were transportation focused. As one participant that worked within a transit focused MP noted:

*“It wasn’t really a specifically articulated goal…transit in general is always an equity conversation. But explicitly we didn’t say this is about enhancing equity for people.”*

Although it is widely advocated to include the principles of equity in the decision-making process, there are not many instances where equity weighs in the formal process [20]. This might be due to a lack of understanding or guiding principles that may aid the stakeholders in operationalizing the concept. For instance, although one of the active transportation plans included examples of neighbourhoods with specific disadvantages, it was not something that guided the partnership in their activities later. A participant noted:

*“I haven’t heard much discussion about that at the table… that social aspect hasn’t really come up from my experience”*

Another participant noted:

*“it’s a subtext. I’d like to think it informs all of our city work, but I know that’s not the case”*

However, participants from food based partnerships said that even though equity was not explicitly discussed, considerations of equity were built-in. Participants seemed to be more confident about the role organizations played to reduce inequities when they framed equity from a ‘food justice’ lens. As one participant stated:

*“Always included always…Our goal and our mandate are to serve everyone, anybody, everybody from the community. So, in every level I would strongly emphasize that that was considered.”*

However, it was apparent that in most of these cases, equity was a part of the larger goal that partner organizations had, and not a specific focus that guided the work of the partnership.

*“Because of what we do and who we are, and the fact that we are a food justice organization is pretty much how we try to design our program. (But) looking at the agreement, I don’t think that was necessarily a huge area of focus.”*

#### Emergent concept

Several participants mentioned that even though the concept of equity has been around for long, explicit focus on equity in program design was still emerging and would get more priority in future works. A participant noted:

*“But I think transit, improving transit always gonna be improving equity…. It was serving a fairly economically vibrant part of the city already but I’m sure that it was beneficial to people of all incomes and backgrounds. Yeah, but it wasn’t central, and I fully admit that, but it has become a very central premise in our work going forward”*

Another participant mentioned:

*“We need to spread around this approach, what can we build from it that is useful and take away from it that’s useful and start to use an equity lens to say where it would be great to do something like this...I think the whole lens of equity is an emerging, it’s always been there in some ways but it’s kind of like in my opinion it’s where the environmental lens was like 10, 20 years ago”*

It was apparent that, considerations of equity mostly came in hindsight. In some cases, success of current or previous partnerships sensitised stakeholders to take more informed decisions in future such as incorporating child- or gender-focused initiatives.

#### Challenges to embed health equity

There were several challenges to embedding equity throughout the processes in multisectoral partnerships. A comprehensive understanding of population health, its determinants, and the equity tenet within it may determine how much consideration is given to equity in program design as well throughout the project life cycle. As one participant noted:

*“It really depends on how you define health like that’s really what it comes down to”*

In addition to this, while talking about the health equity lens, participants mentioned being comfortable with terms such as ‘poverty’ and ‘living wages’ as opposed to equity. A participant mentioned:

*“It’s much easier to develop, to talk about the importance of local food, to talk about food culture, to talk about all of those sorts of I mean they’re important. They’re important and big picture, they all contribute towards a stronger food system but they’re much easier to talk about. Poverty and, poverty as it relates to the food system and thinking about people’s access to food for financial reasons - it’s much easier to talk about those other things.”*

What is important to note in these discussions is the gap that lies between understanding and acting on equity conversations and the language used to orient action. None of the participants mentioned addressing the social structures or the social determinants of health and inequity in the conversations. It’s also important to acknowledge that the gap that lies between organizational preference and actual work that multisectoral partnerships put forward. For example, although alignment of goals was found to be critical for the success of multisectoral partnerships, participants view on how those goals could be attained differed. At times, participants seemed unsure of the initiatives taken, even though equity was a determinant factor in organizational priority setting as well as partnership formation. Designing intervention in a way that appeals to all related stakeholders is critical. Inability in doing so limits smooth collaboration leaving a negative impact on the partnership outcome.

#### Style of leadership

Overall, the collaborative management style, provided in a structured and formal manner, enabled stakeholders to have a clearer idea of roles and responsibilities that were also conducive to navigate partnership-based works more efficiently. Leaders were found to be the key actors in bringing equity into partnership dynamics. Types of leadership in multisectoral partnerships played a crucial role in keeping the organization’s work flowing within organizational boundaries as well as with other partners. A transit focused multisectoral partnership had leadership that occurred through a core group of members involving all the stakeholders. One participant from a transit focused partnership stated:

*“What we did that was really helpful was we set up sort of a leader’s table which was the Heads of the different divisions who were viewed as co-leading the project.”*

This kind of collaboration enabled sharing of responsibilities and easier decision making. It was considered an important factor for sustaining coalition effectiveness, maintaining progress, and bringing the required changes. The democratic and collaborative decision-making process, i.e., a partnership process where each partners opinions were weighed in, was seen to increase satisfaction among members of the organization. The participant further mentioned:

*“One of the reasons why setting up the leader’s table was so important was to have a venue or a forum for those opinions to be voiced and decisions to be made. There were different opinions along the way and I think just like any big initiative we worked them out and I think that actually served really well as a model for making sure that there is a forum or a process set up for decision making and conflict resolution.”*

Participants noted that a formal, yet collaborative decision-making process made it easier to be heard. Development processes became more transparent when there was a collaboration between leaders as compared to the autocratic form of leadership where decision making power was at the discretion of only one partner. Interviewees noted that it was helpful and pleasant to work with partnerships that were more progressive and where leadership was distributed among stakeholders. One participant expressed concern and dissatisfaction about organizational hierarchy. The participant said that traditional top-down leadership, which was based on authority and position did not leave room for smaller partner organizations to feel supported:

*“There’s a desire to control at the top so when you reach out to others across the organization, across the city in other departments, it’s not supported, it’s a little, seems a little threatening or it has to get approval from people above and it just can’t work like that.”*

Along with the challenges that existed in a top-down leadership such as a disconnection in collaborative decision making and working, there still remained a pressure to deliver program objectives. One participant noted:

*“I think their relationship has been like: we give you the money and you get the money, and we just want you to tell us that this is what you’ve done.”*

#### Core competencies of a leader

##### Communication

Across partnerships, it was implicit that an effective leader in public health must have core competencies such as communication, negotiation, problem-solving, and an ability to embed equity throughout the program for effective outcomes. Participants mentioned working together was vital and clear and open discussions helped in overcoming miscommunications and conflicts.

*“We were able to work together, just having very open and transparent discussions helped us to overcome that and if there was a conflict, work together in order to resolve it.”*

A participant from another partnership however said,

“*We just want the number. We don’t want to hear about your thing… there might be better ways for us to do things; if we are all willing to have those tough conversations, but sometimes some of us are not”.*

It is considered important that the leaders within partnerships are willing to have conversations regarding key decision-making processes such as funding and are willing to listen to feedback from the partnership that is being funded.

Participants mentioned that projects dealing with social determinants of health took a longer time to demonstrate the expected outcomes and it was difficult to negotiate and secure funds for such issues due to their complex nature. It required leaders to cast these issues in a way that would facilitate securing funds. As leaders from one partnership opted for “*dress it up*” approach to gain the required attention for the “*less sexy stuff*”, one participant said,

*“let’s find an alternative for the kind of, the less sexy stuff that is absolutely fundamental but for whatever reason just doesn’t resonate... we were always trying to dress it up well not dress it up but just sort of package it in a way…and articulate it ‘cause it’s complex and it’s difficult in its very nature.”*

Different from above, leaders who possessed the ability to communicate and negotiate effectively were more successful in mobilizing funds and ensuring equitable opportunities for all citizens. An example noted by one participant:

*“So, she negotiated with that team to get some of that money to develop a program that would accommodate low-income folks who were interested in getting their food safety training and certification.”*

##### Taking ownership

Participants expressed concern and dissatisfaction with partners and their leaders for not owning up to the delivery and implementation of actions. For example, one participant noted:

*“it’s kind of floating back and forth but no one’s owning it”*

Participants, particularly from food partnerships, mentioned that the issue of lack of leadership and ownership arose partly due to food being a diverse and complex phenomenon that could be approached from various sectors such as environment, sustainability, health, or interest in local foods and local food culture. Lack of leadership in terms of taking responsibility made it more difficult to collaborate and decide on how to accomplish the outcomes. A participant noted:

*“I think in the short term it makes it very hard to coordinate because you can’t point to any one person or any one group and say you’re responsible for ensuring the people ... have good food. Because … I don’t think there’s any organization or person that feels that’s their responsibility.”*

##### Networking

Participants stressed the importance of having different leaders come together and work towards solving new problems, sharing data, and making critical decisions. Being able to build relationships with fellow leaders was thought to be an important characteristic of an effective leader in projects involving multiple organizations.

*“Without the strong leadership it just doesn’t work, none of this stuff works so that’s what we’re really trying to bring and continue going forward is when we have these big flag ship projects or major initiatives we really need to engage our leaders and have them really be more hands-on of what some of the key decision points are..”*

The participant also added,

*“it can’t just be we all brief everybody independently and everyone’s okay, it’s getting them together in a room physically and building that relationship so that they can be more effective leaders collectively than independently…the second point which is just about collaboration in general, so starting with collaboration at the top with the leaders, seeing that cascade down I think is really important.”*

Pre-existing relationships were seen to build sustainable partnership bonds. Leaders coming from a place of trust and historic friendship were considered as enablers in steering forward an effective partnership.

*“it’s very clear to me that interpersonal relationships play a huge role in how these multi-sectoral partnerships are successful. How they’re implemented, how they’re successful, the most successful ones are the ones that really rely on personal relationships.”*

### Quantitative survey findings

Around 48% of respondents felt that one of the strengths in their partnership was bringing in all the essential partners whenever needed. Around 58% respondents said that points of views from all the partners were given equal importance in discussions and decision making. Around half (50%) of the participants felt that their partnership was able to acknowledge contributions made by each partner equally, and that partners were also benefitted equally from the partnership. Across Saskatoon and Toronto, only around one-tenth of respondents stated accountability to be a strength in their multisectoral partnership. Less than half of respondents from MPs mentioned that benefits were distributed equally, and diverse points of view were given equal consideration in decision making.

### A synopsis of the organizational mission and vision statements

Table 2 shows a summary of the partnership goals. Of the six MPs that we studied, five did not make any direct and explicit reference to equity in their goals or program aims document. Only one multisectoral partnership expressed interest to bring social and economic equity. Food focused MPs either made reference to equity seeking groups such as ‘low-income neighborhoods’ or ‘people with disability’ or mentioned working towards reducing inequality in their program goals; but no mention of health equity was found.

**Table 2:** Summary of goals of the multisectoral partnerships located in Toronto and Saskatoon

Multisectoral partnerships (MP)	Summary of partnership’s goals/ mission & vision statement
MP-Health #1	This MP was formed with a specific focus on helping newcomers to settling in Canada in 2016. The aims and goals of the MP mentioned bringing social and economic equity to the lives of participants. The intervention was tailored to specific groups of people that needed the intervention most
MP-Health #2	This partnership was formed in 2011 and the partnership strategy was outlined in 2014. It focused on improving and connecting inner city neighbourhoods. The strategy had multiple recommendations and goals, however none of them made any explicit reference to equity, or health equity in particular
MP-Food #3	This MP was formed with a specific focus to promote access to good food. The program goals aimed at reaching low-income neighborhoods as well as neighborhoods having least access to transportation services and food stores. This MP did not make any direct reference to ‘equity’ or ‘health equity’ in its program goals, however, the goals were oriented around equity-seeking groups such as ‘low-income neighborhoods’, and people not having ‘adequate transit services’
MP-Food #4	This MP was formed in 2012 with goals of ensuring food access for everyone, enriching local food culture and minimizing ecological footprint. It specified several recommendations, one of which was ‘reducing inequality’
MP-Active Transit #5	This transportation focused MP did not mention health equity in its program aims and objectives. However, it mentioned assessing the impacts of the project, including safety of vulnerable people as well as accessibility to all
MP5-Active Transit #6	This MP was formed in 2015 to promote active transportation. Goals and targets of the partnership mentioned improving connectivity, safety, convenience etc., however, none of the goals or action targets mentioned health equity

## Discussion

The purpose of this study was to investigate how multisectoral partnerships promoting healthy eating and active living conceptualized and attained equity in their work. We used the Lambton framework which posit that internal equity refers to seven elements that are internal to the partnerships such as ‘the processes, knowledge and resources of an organization that enables the partnership to attain its goals’ [19]. The internal elements influence organizational capacity to attain health equity in three key levels-individual, organizational, and structural. These levels of influences are interdependent, and according to the Lambton framework, the individual level has a relatively smaller amount of influence to exert compared to organizational and structural levels. However, while examining some of these key elements through our study, it has become apparent that the individual level is perhaps the most crucial of the three-capable of inducing changes in partnerships to a greater extent than thought before. Our findings indicate that individual construction of equity determines the extent of priority equity gets as a cornerstone practice in multisectoral partnerships. Therefore, a leader who has clarity over the concept of equity and its antecedents will most likely facilitate equity conversation among the partners, which will then be reflected in the partnerships mission and vision statements, work plans, and interventions. Therefore, it is significant to understand how equity is being perceived and prioritized by people who upholds the responsibility to attain it through their collective actions. This also refers to the need of including youth in equity conversations in their formative years. Because, achieving equity in multisectoral partnership is a combination of strengthening both individual skills, understandings, and organizational capacity [19].

A barrier to attaining equity within and between partnerships across MPs located in Saskatoon and Toronto, was that most participants did not have a shared understanding of health equity and how the concept can be acted upon. This is consistent with previous study findings that found a lack of collective understanding of health equity [21, 22]. In our study, participants identified health equity around geographic accessibility mostly, and hence programs were designed to make services physically accessible to people such as designing a bike lane or introducing mobile markets. Participants rarely mentioned socio-economic factors such as income and education, or crucial social processes such as racism or sexism. Therefore, participants’ construction of health equity around geographic accessibility only has the risk of decontextualizing how certain population groups are placed differently across social hierarchies, i.e., the conditions that created social inequities in the first place [22]. Inability to acknowledge how policy impact differs by these hierarchies limits capacity of multisectoral partnerships to bring optimum levels of health outcome for all [16].

The extent of priority that equity is given during project initiation as well as in regular activities varied with the type of partnership. Most participants from food focused MPs mentioned considering equity in their program design, while participants from transit focused MPs mentioned that employing equity lens in program design is still emerging. Transportation focused MPs were also more inclined towards a place-based notion of equity whereas food focused MPs were more associated with reducing gaps in food access; a basic need according to Maslow’s hierarchy of needs [23]. Food, as a basic need might be a reason why food focused MPs seemed to be more closely aligned with the essence of equity compared to the transit focused ones. In addition to this, on analyzing the goals/targets of the multisectoral partnerships as mentioned in their official documents, we found that only one among the six MPs explicitly mentioned equity while a few mentioned ‘low-income neighbourhoods’. Similar to the findings of Pauly et al., discussions on intersectionality and underlying social structures that is liable for making some communities more vulnerable compared to others were absent [22].

Several studies found leadership to be an essential component in multisectoral partnerships as it brings synergies between organizations [24]. Traditional literature on leadership styles in public health domain has emphasized mostly on top-down versus bottom-up leadership styles, with a connotation that ‘top-down’ leadership style is ill suited for collaborative efforts [25]. Often in top-down leadership style, designated leaders design programs with explicit implementation strategy having limited opportunities for community partners to contribute [25, 26]. On the contrary, in a bottom-up approach community stakeholders are key in program planning and designing [26]. However, as argued by Koontz et al. in reality these differences might not be distinct at all. For example, if government becomes a part of collaborative multisectoral partnerships, certain elements of their legislative structure can become elements of the partnership itself [25]. Therefore, collaborative leadership can be either top down or bottom up, and a dynamic leader can facilitate a balance between these complexities [25]. Our study sheds light on these issues. In multisectoral partnerships across Saskatoon and Toronto, the style of leadership emerged as a key factor that influenced bringing equity in program design and resource mobilization. Around half of participants emphasized the need of appreciating varied opinions and acknowledging efforts. Collaborative leadership was found to be more efficient to maintain equity in partnership dynamics compared to authoritative approaches. Formal decision-making processes enabled leaders to resolute conflicts more easily. Core competencies of a leader that emerged to be pivotal in sustaining synergies within and beyond partnerships were the leader’s skills to communicate and negotiate effectively, be inclusive and transparent in the decision-making process, and utilize pre-existing relationships to navigate partnership challenges.

Our study further identified challenges faced by MPs in employing an equity lens in partnership-based works. Participants’ definition of population health determined how equity gets embedded in the decision-making process. Stakeholders acknowledged being comfortable using generic terms. Evidence from our study corresponds to similar findings in other settings. For example, in a study done with the public health leaders of British Columbia, Pauly et al. found that equity was constructed around accessibility to health care services, and participants mostly used proxy terms to indicate health inequity [22].

An analysis of national nutrition policies of the high-income countries including Canada revealed that majority of government policy documents highlighted the need to develop multisectoral partnerships to combat diet related health inequities [27]. However, even though equity was mentioned to be a driving principle in these documents, proposed policy actions were often unclear of how equity would be attained [27]. Henson et al. found that senior health officials often felt health equity to be a politically heavy concept—sometimes even difficult to comprehend [28]. Another study found a paucity of programs addressing healthy eating and physical activity in Canadian provinces like Ontario and British Columbia that included structural interventions-a key to eliminate socio-economic inequities [29]. Our findings represent important indicators that can enable multisectoral partnerships to have fluid and explicit conversations about incorporating and advocating health equity in all phases of program development and implementation. The health and social inequities are widening, and this gap has been intensified more than ever before with the COVID-19 pandemic [30]. To attain success in reducing health inequities, of foremost importance is that equity should be the common theme that binds the work of partnerships. Stakeholders should make effort to understand the interconnectedness of the various fields and how the quality of their collaboration can impact the outcomes. Achieving health equity in a short span of time is also difficult, therefore, MPs should consider this to be a process over time. It is also necessary that multisectoral partnerships conduct equity analysis to assess its reach, impact, and contexts of the impacts [31].

Health inequities are complex and multifaceted. Therefore, a need for collaborative engagement from multiple entities arises [32]. Because partners contribute resources and expertise differently, multisectoral partnerships often have a hierarchy in their work. Hence, equity in partnership also refers to an arrangement where all partners have equal opportunities to take part to define problems, design solutions, and have equal voices in implementation [33]. Partnerships that uphold the value of equity within the decision-making process are believed to have a stronger impact on the community. However, lack of common understanding of health equity across partners can lead to having varied visions of partnership goals and how equity can be attained both within and beyond the partnerships. The various internal drivers identified in our study were interdependent. These internal drivers are influenced by external drivers such as health policies, resources, and politics as well [19]. However, studying the external drivers such as politics and funding were beyond the scope of this study. Nevertheless, it is an interesting area that future research should look in to get a complete understanding of the role of multisectoral partnerships towards attaining health equity, and how the various factors influence this process.

## Conclusion

This study adds to the evidence base that identifies key internal factors of multisectoral partnerships to attaining health equity goals across the populations they serve. The findings suggest that attention should be paid to how different partners comprehend health equity. To accomplish optimal functioning of multisectoral partnerships, partnerships should monitor if their current leadership styles and competencies are efficient and can bring the required output and sustainable success. Attaining consistency in defining what equity means and commitment to attaining it within the mandates of multisectoral partnerships to achieve their population health goals have yet more work to do.

## Data Availability

The datasets used and/or analyzed during the current study are available from the corresponding author on reasonable request.

## Abbreviations

MUSE: Multisectoral Urban Systems for health and Equity in Canadian cities
WHO: World Health Organization
SETAP: Self-evaluation Tool for Action in Partnership
MP: Multisectoral partnership
HiAP: Health in All Policies

## Footnotes

1 A short description of the six multisectoral partnerships examined in this study is provided in Table 1 of the results section.

**Table 1:** Strengths of multisectoral partnerships

Strengths	%
All the essential partners were included in the partnership	48
All points of view are given equal consideration	58
Everyone’s part in carrying out activities is acknowledged equitably	50
Partners benefit equitably from the partnership	50
Accountability	10
Offer programs in varied living environments	19
